# Increased FGF21 plasma levels in humans with sepsis and SIRS

**DOI:** 10.1530/EC-13-0040

**Published:** 2013-09-17

**Authors:** Karim Gariani, Geneviève Drifte, Irène Dunn-Siegrist, Jérôme Pugin, François R Jornayvaz

**Affiliations:** 1 Service of Endocrinology, Diabetes, Hypertension and NutritionGeneva University HospitalsRue Gabrielle-Perret-Gentil 41211, Geneva 14Switzerland; 2 Laboratory of Intensive CareGeneva University HospitalsRue Gabrielle-Perret-Gentil 41211, Geneva 14Switzerland; 3 Department of Microbiology and Molecular Medicine, Faculty of MedicineUniversity of Geneva1211, Geneva 14Switzerland

**Keywords:** growth factors, metabolism, inflammation

## Abstract

Fibroblast growth factor 21 (FGF21) is a key regulator in glucose and lipid metabolism and its plasma levels have been shown to be increased not only in humans in different situations such as type 2 diabetes, obesity, and nonalcoholic fatty liver disease but also in animal models of sepsis and pancreatitis. FGF21 is considered as a pharmacological candidate in conditions associated with insulin resistance. The aim of this study was to compare FGF21 plasma levels in patients with sepsis, in patients with systemic inflammatory response syndrome (SIRS), and in healthy controls. We measured FGF21 plasma concentrations in 22 patients with established sepsis, in 11 with SIRS, and in 12 healthy volunteers. Here, we show that FGF21 levels were significantly higher in plasma obtained from patients with sepsis and SIRS in comparison with healthy controls. Also, FGF21 levels were significantly higher in patients with sepsis than in those with noninfectious SIRS. FGF21 plasma levels measured at study entry correlated positively with the APACHE II score, but not with procalcitonin levels, nor with C-reactive protein, classical markers of sepsis. Plasma concentrations of FGF21 peaked near the onset of shock and rapidly decreased with clinical improvement. Taken together, these results indicate that circulating levels of FGF21 are increased in patients presenting with sepsis and SIRS, and suggest a role for FGF21 in inflammation. Further studies are needed to explore the potential role of FGF21 in sepsis as a potential therapeutic target.

## Introduction

Fibroblast growth factor 21 (FGF21) is a member of the FGF superfamily, consisting of FGF19, FGF21, and FGF23 (1, 2, 3, 4). Because they lack the conventional FGF-heparin-binding domain, these FGFs can escape the body's vast deposition of heparansulfate proteoglycans and can be released into the circulation and function as endocrine factors (5). FGF21 is a hormone mainly produced by the liver and white adipose tissue, where it is in part induced by the peroxisome proliferator-activated receptor α (PPARα) and PPARγ respectively (1, 6). FGF21 has emerged as a key regulator in the metabolism of glucose and lipids (6, 7, 8). FGF21 may have a potential role as a therapeutic agent for conditions associated with insulin resistance as it has been shown that administration of a recombinant form of this hormone in obese mice and diabetic monkeys improves insulin sensitivity, body weight, and lipid profile (9, 10, 11, 12, 13, 14).

Sepsis represents a continuum beginning with a host–pathogen interaction that triggers a complex interplay between pro-inflammatory, anti-inflammatory, and apoptotic mediators. Mortality estimates vary, but severe sepsis and septic shock carry high mortality rates, possibly up to 50% (15, 16). The number of sepsis-related admissions in intensive care units (ICU) has been steadily increasing over time (17, 18). Insulin resistance is a common feature in sepsis and is due to several metabolic alterations (19, 20, 21, 22, 23, 24). Biomarkers play an important role in different clinical situations including sepsis (25, 26, 27). Certain biomarkers, such as procalcitonin (PCT), may indicate the presence or absence of sepsis and its severity. Moreover, some biomarkers, such as PCT, may have other potential roles including the differentiation of bacterial infection from viral infection, the assessment of the response to therapy, or the prediction of the development of multiple organ failure (25, 26, 27). However, the precise role of biomarkers in the management of septic patients remains to be defined (25, 26, 27).

A possible role of FGF21 in sepsis has been suggested by the observation of increased circulating levels of this hormone during experimental sepsis in mice. Moreover, its administration has a protective effect from the toxicity of lipopolysaccharide (LPS) and sepsis (28). FGF21 can also reduce the severity of cerulein-induced pancreatitis in mice (29), further indicating that FGF21 could modulate inflammation. These findings highlight the possible role of FGF21 as a biomarker and a therapeutic tool in mice with sepsis and an inflammatory state, leading us to explore its role in humans with sepsis and systemic inflammatory response syndrome (SIRS) as compared with healthy subjects. The aim of this study was therefore to evaluate the circulating plasma levels of FGF21 in patients with sepsis, SIRS, and in healthy control subjects. We also assessed potential correlations between FGF21 and scores of severity of sepsis as well as clinical outcomes.

## Subjects and methods

### Patients

Adult patients (>18 years) hospitalized in the ICU from a single university hospital (36 beds, mixed surgical and medical ICU, 3400 admissions/year) between March 2010 and November 2011 were enrolled in the study if they presented with severe sepsis, septic shock or noninfectious SIRS within 24 h after admission. Sepsis and SIRS were defined according to current definitions (30, 31). Clinical characteristics, the acute physiology and chronic health evaluation II (APACHE II) score, and the simplified acute physiology score 2 (SAPS2) were also collected (32, 33). All patients were treated following evidenced-based medicine guidelines. The study was conducted in accordance with the Declaration of Helsinki and the International Conference on Harmonization Guidance for Good Clinical Practice and was approved by our institutional Ethics Committee.

### Blood analyses

Blood samples were obtained from each patient upon admission. Twenty milliliters of heparinized blood were drawn from patients via the arterial catheter, and by venous puncture in healthy volunteers. Plasma FGF21 concentrations were measured using a commercial ELISA Kit (BioVendor GmbH, Heidelberg, Germany). C-reactive protein (CRP), PCT, and glucose were measured using standard methods by the central laboratory of Geneva University Hospital.

### Hepatocytes stimulation

HepG2 and HUH7 hepatoma cells lines (ATCC, Manassas, VA, USA) were seeded into a 96-well plate to confluence. Glucose (25 mM), interleukin 1β (IL1β; 1 ng/ml), IL6 (10 ng/ml), and glucocorticoids (10 μM) were added to the cells for 24 and 48 h in a serum-free DMEM/F12 medium. Supernatants were collected and FGF21 was measured batchwise by an ELISA Kit (BioVendor GmbH). Cell stimulation experiments were performed in triplicate.

### Statistical analysis

Data are presented as means±1 s.d. Differences between groups were tested using ANOVA with Bonferroni's adjustment for multiple comparisons and correlation coefficients given are Spearman's (GraphPad Prism 6). A *P* value <0.05 was considered statistically significant.

## Results

### Patients' characteristics

Forty-five subjects were enrolled into three groups: 22 patients with sepsis, 11 patients with noninfectious SIRS, and 12 healthy volunteers. Age and BMI were significantly lower in healthy controls than in patients with sepsis. Clinical characteristics are summarized in Table 1. Among patients with sepsis, five had type 2 diabetes (three were treated with oral antidiabetic agents alone, one with insulin alone, and one with both). Among patients with SIRS, two had type 2 diabetes and were treated by oral antidiabetic agents alone. Leading sources of infection in septic patients were the respiratory tract, the intra-abdominal cavity, and the urinary tract. Bacterial cultures (from blood, urine, or bronchial aspiration) revealed *Streptococcus pneumonia* (5 patients), *Escherichia coli* (3), *Enterococcus faecium* (1), *Enterococcus faecalis* (1), *Haemophilus influenza* (1), *Proteus mirabilis* (1), or *Klebsiella pneumonia* (1). No bacteria were found in nine patients. Patients with noninfectious SIRS were admitted for trauma (4 patients), hemorrhagic choc (2), alcohol-related pancreatitis (1), HELLP syndrome (1), pulmonary embolism (1), coronary artery graft surgery (1), or postoperative respiratory failure (1).

### FGF21 levels in patients with sepsis, SIRS, and in healthy controls

**Figure 1:**
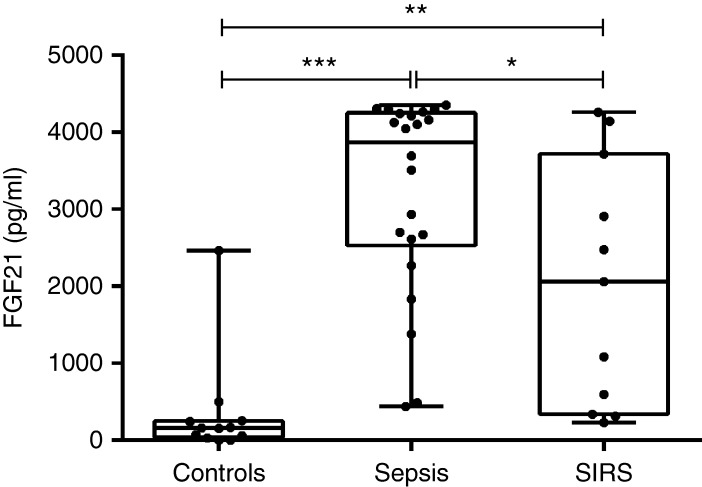
FGF21 plasma levels in healthy controls, patients with sepsis, and patients with SIRS. Box and whiskers plot show all values, from minimum to maximum. Data are means±s.d., *n*=11–22. *P<0.05, **P<0.01, and ***P<0.001.

FGF21 serum levels were increased by approximately tenfold in patients with sepsis compared with healthy controls (Fig. 1). We also observed an approximately sixfold increase in FGF21 levels in patients with SIRS compared with healthy controls (Fig. 1). FGF21 was found to be significantly higher in patients with sepsis compared with those with noninfectious SIRS (Fig. 1). As healthy volunteers were significantly younger than patients with sepsis, we performed a subgroup analysis, considering only older controls (*n*=6; mean age 49.3±7.4 years, *P*=NS vs sepsis; BMI 22.3±2.8 kg/m^2^, *P*=NS vs sepsis). In these conditions with matched age and BMI, plasma FGF21 levels remained significantly increased in patients with sepsis compared with healthy controls (523±954 pg/ml in healthy controls, *P*<0.001).

### Relationship between FGF21 levels, severity of sepsis, and biomarkers of inflammation

**Figure 2:**
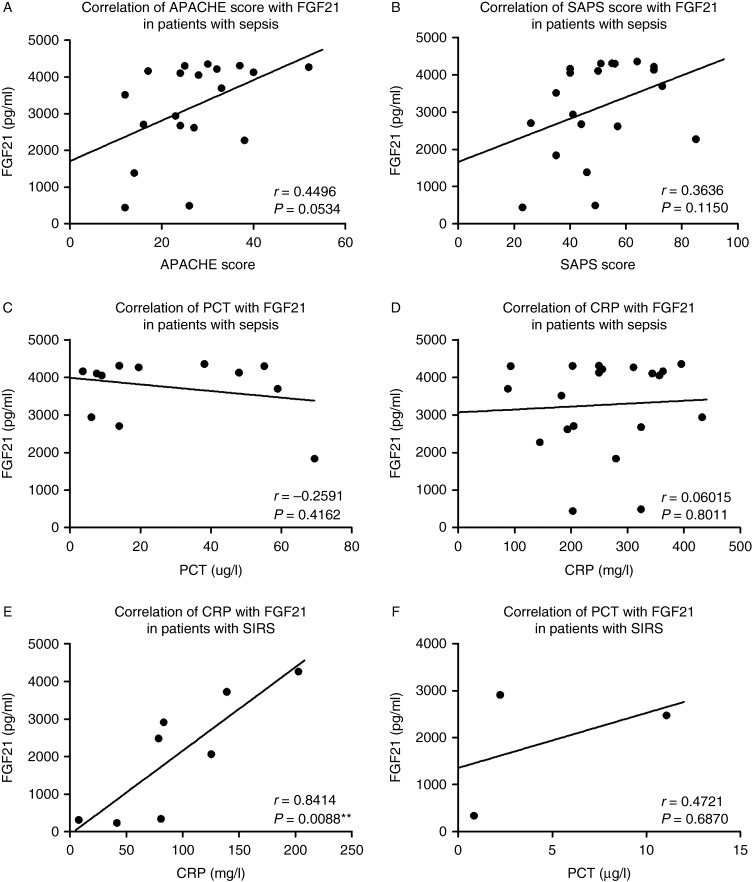
Correlations between FGF21 in sepsis or SIRS and different markers/scores of sepsis and SIRS. (A) FGF21 and APACHE II score in sepsis, (B) FGF21 and SAPS2 score in sepsis, (C) FGF21 and PCT in sepsis, (D) FGF21 and CRP in sepsis, (E) FGF21 and CRP in SIRS, and (F) FGF21 and PCT in SIRS. Correlation coefficients are Spearman's, *n*=3–20, **P<0.01.

The severity of sepsis was evaluated upon admission by the APACHE II and SAPS2 scores. There was a positive correlation between FGF21 levels on admission and APACHE II score, at the limit of statistical significance (*P*=0.053; Fig. 2A). FGF21 levels did not significantly correlate with SAPS2 score, although we found a positive trend (Fig. 2B). In patients with sepsis, we did not find any significant correlation between plasma levels of FGF21 and PCT (Fig. 2C), a specific marker of sepsis, or with CRP (Fig. 2D). In contrast, in patients with SIRS, there was a strong positive correlation between FGF21 and CRP (Fig. 2E), but not with PCT (Fig. 2F).

### Time-course of FGF21 during sepsis

**Figure 3:**
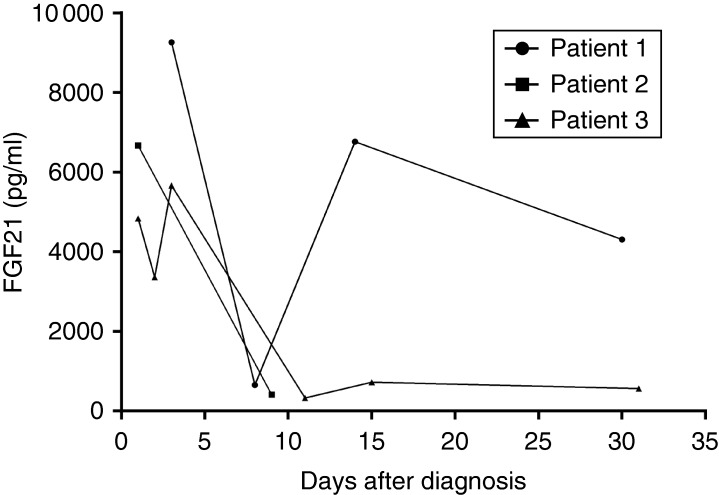
Time-course of FGF21 in three different patients with sepsis. Patients 1 presented a nosocomial pneumonia around day 10, which resolved after appropriate antibiotic administration.

We analyzed serial samples of FGF21 in three patients with sepsis. Plasma levels of FGF21 decreased for all patients during the follow-up and paralleled the improved clinical condition, except in one patient (patient 1, Fig. 3). For this patient, the rise in FGF21 levels after a first descent could be attributed to an episode of nosocomial pneumonia developed during the ICU stay. After antibiotic treatment, FGF21 levels decreased in the days following.

### Is FGF21 an acute-phase protein?

To study the possibility that FGF21 could be an acute-phase protein (APP), HepG2 and HUH7 hepatoma cell lines were cultured in the presence of different cytokines (IL1β and IL6) known to stimulate the production of APPs (26, 31, 34). After 48 h, levels of FGF21 were determined by ELISA in culture supernatants. HepG2 and HUH7 cells were selected because they are widely used to study the production of APPs. However, we were unable to detect FGF21 in conditional supernatants from hepatoma cell lines stimulated with pro-inflammatory cytokines (data not shown). These data suggest either that FGF21 cannot be considered as an APP or that we were below the detection threshold of the ELISA.

## Discussion

Sepsis is accompanied by glucose and lipid alterations, and FGF21 is considered as a key regulator in glucose and lipid metabolism (1, 6, 7, 8, 11). FGF21 plasma levels are increased in type 2 diabetes, obesity, nonalcoholic fatty liver disease (NAFLD) and dyslipidemia (35, 36, 37, 38, 39), but injection of FGF21 in animal models of insulin resistance has been shown to improve this condition (9, 10, 11, 13, 14). Therefore, FGF21 can be considered as a pharmacological candidate useful in these conditions. Interestingly, FGF21 has also proven to be beneficial in conditions associated with inflammation such as pancreatitis or sepsis in animal models (28, 29), suggesting a role of FGF21 in modulating inflammation.

In this study, we show for the first time that patients with sepsis have a significant elevation of FGF21 plasma levels, higher than patients with noninfectious SIRS or healthy subjects. Patients with SIRS had significantly higher FGF21 plasma levels than healthy controls. These results confirm findings in endotoxemic mice where increased FGF21 levels were found in plasma (28). Interestingly, we did not find any significant association between FGF21 upon admission and PCT, a specific biochemical marker of sepsis, or with CRP. This can be due to differences in kinetics between FGF21, PCT, and CRP. However, we found a strong positive association between FGF21 and CRP in patients with SIRS that may be explained by a similar kinetic increase of these two proteins in a situation with less inflammation than that found in septic patients. The second hypothesis may be that CRP and FGF21 are less specific, and are both increased in inflammatory states not linked to sepsis as opposed to PCT, which has been shown to be more specific for sepsis than CRP (34, 40).

We also noticed that the evolution of FGF21 plasma levels using serial samples from patients with sepsis was correlated with their clinical evolution, with a decreasing plasma level associated with clinical improvement. Furthermore we found, in one patient with sepsis who presented with a second infection during the stay in ICU, an initial decrease followed by an increase in FGF21 levels paralleling the nosocomial infectious episode. This highlights the fact that FGF21 may be considered as a biochemical parameter of follow-up in sepsis and a possible marker of recurrence. However, the exact kinetic of FGF21, particularly in the situation of sepsis, needs to be further explored to better define its role in this clinical situation. Finally, we found a weak positive association between FGF21 serum levels and the severity of sepsis according to the APACHE II score, but not with SAPS, meaning that FGF21 cannot be considered as a good marker of clinical severity in adults admitted in ICU for sepsis or to assess the risk of mortality.

Consistent with our findings, a recent report has shown a twofold increase in FGF21 plasma levels in mice induced after the administration of LPS, zymosan, and turpentine. However, this increase in FGF21 was not the result of an increased expression of FGF21 in the liver as hepatic FGF21 mRNA was decreased. Therefore, in this case, increased FGF21 levels might be secondary to increased synthesis of FGF21 by extrahepatic tissues such as white adipose tissue and skeletal muscle (28). As FGF21 plasma levels increase after sepsis induction in mice, potentially as a counter-regulatory response, it is tempting to hypothesize that FGF21 administration could have the potential to modulate inflammation. Indeed, treatment with exogenous FGF21 reduced the rate of death and the rapidity of death after endotoxemia, suggesting that the increase in plasma FGF21 during an inflammatory state may be a protective response (28). Another study has shown that in mice with cerulein-induced pancreatitis, the expression of FGF21 increased rapidly and dramatically (29). Moreover, this study highlighted that FGF21 is an immediate response gene during acute pancreatic injury that could stimulate a autocrine/paracrine-signaling phenomena to reduce tissue inflammation and fibrosis. Our results are therefore consistent with these results in the fact that FGF21 can be considered as a marker of the presence and also the severity of cellular injury present in situations of inflammation such as acute pancreatitis, SIRS, and septic shock.

However, as sepsis is associated with insulin resistance, we can also hypothesize that the increase in plasma FGF21 observed in subjects with sepsis and SIRS might also be due, at least partly, to insulin resistance. Insulin resistance in sepsis is due to a decreased effect of insulin, but also reflects an imbalance between insulin and its counter-regulatory hormones (cortisol, glucagon, growth hormone, and catecholamines) (19, 21, 22, 23, 24). This results in acute hyperglycemia that may participate in the maintenance of an inflammatory response (22). As high insulin sensitivity has been shown to have a good performance in ruling out sepsis, FGF21 may be a good tool to exclude sepsis in suspicious cases (20).

We could not detect the presence of FGF21 in supernatants from HepG2 and HUH7 cells after stimulation with IL1β, IL6, and high glucose concentrations. This can mean either that FGF21 cannot be considered as an APP or that the ELISA test was below the detection threshold. However, the ELISA test is supposed to be used with plasma, where concentrations are supposedly much higher than in cell supernatants. Finally, it could actually have been that our cell experiments would have resulted in a decrease in FGF21 expression in HepG2 and HUH7 cells. Consistently, others failed to stimulate FGF21 expression in 3T3-L1 adipocytes with cytokines or LPS and were also unable to inhibit FGF21 expression in Hep3B cells with cytokines (28). FGF21 is not a specific biomarker of sepsis because it is also increased in patients with SIRS. However, because both clinical situations consist of a widespread inflammatory response with a continuum between SIRS, sepsis, severe sepsis, and septic shock, the higher FGF21 levels in patients with sepsis than with SIRS suggests that FGF21 could be a marker of the severity of the inflammatory response. Moreover, if one considers that APP can be synthesized outside the liver, then FGF21 could be considered as an APP.

Although we could show a potential role of FGF21 in sepsis and SIRS, our study has some limitations. It was retrospective and as a consequence included a relatively limited sample size with some degree of heterogeneity and missing values. Notably, age and BMI were not similar among the three different groups, patients with sepsis being the oldest and healthy controls being the heaviest. As age and BMI are known to affect FGF21 levels (39), the absence of matching for these parameters among the three groups may have influenced our results. However, our subgroup analysis where age and BMI were matched between patients with sepsis and healthy controls showed that FGF21 plasma levels remained significantly increased in patients with sepsis. Owing to the small sample size, we were not able to correct the values for confounding factors also known to increase FGF21 levels, such as type 2 diabetes or NAFLD (35, 36, 37, 38, 39). However, we think that the levels of FGF21 that we measured in sepsis and SIRS were much higher than those found in the aforementioned conditions associated with insulin resistance, and clearly suggest a role of sepsis and SIRS *per se* in FGF21 elevation, consistent with studies on animals (28, 29). Also, our study did not address the causal relationship between FGF21 and sepsis. Therefore, further prospective studies are warranted to determine whether elevated FGF21 is directly caused by sepsis or is a marker of the insulin resistance present during sepsis. Moreover, it will be important to determine which is the major source of FGF21 production in these conditions and if FGF21 could have the potential to modulate inflammation in humans.

In conclusion, this study is the first to report a systemic release of FGF21 in human sepsis and SIRS, with plasma levels markedly higher than those found in healthy controls. Further studies are needed to better delineate the role of FGF21 in conditions associated with inflammation such as sepsis and SIRS. FGF21 could, however, represent a novel potential therapeutic target in these conditions and its potential role in modulating inflammation warrants further research.

## Author contribution statement

K Gariani designed experiments, researched data, and wrote manuscript. G Drifte researched data and contributed to discussion. I Dunn-Siegrist designed experiments, researched data, and contributed to discussion. J Pugin designed experiments, contributed to discussion, and reviewed/edited manuscript. F R Jornayvaz designed experiments, researched data, reviewed/edited manuscript, contributed to discussion, and wrote manuscript. F R Jornayvaz is the guarantor of this work and, as such, has full access to all of the data in the study and takes responsibility for the integrity of the data and the accuracy of the data analysis.

## Figures and Tables

**Table 1:** Clinical characteristics of patients with sepsis, noninfectious SIRS, and healthy volunteers. Data are shown as means±s.d. (*n*=3–22).

	**Sepsis**	**SIRS**	**Healthy controls**
*n*	22	11	12
Gender	5 F, 17 M	2 F, 9 M	6 F, 6 M
Age (years)	65.2±17.9^†^	55.0±15.8	39.5±12.6
BMI (kg/m^2^)	27.3±7.4*	25.1±4.3	21.6±2.8
APACHE II	26.6±10.2	20.6±6.1	NA
SAPS	51.5±16.4	47.6±14.0	NA
PCT (μg/l)	28.1±22.9	4.7±5.6	ND
CRP (mg/l)	263.1±95.4^‡^	94.8±60.4	ND

F, female; M, male; NA, not applicable; ND, not determined. *P<0.05 and ^†^*P*<0.001 vs healthy controls. ^‡^*P*<0.001 vs SIRS.
